# Neurogenesis in zebrafish – from embryo to adult

**DOI:** 10.1186/1749-8104-8-3

**Published:** 2013-02-21

**Authors:** Rebecca Schmidt, Uwe Strähle, Steffen Scholpp

**Affiliations:** 1 Karlsruhe Institute of Technology (KIT), Institute of Toxicology and Genetics (ITG), 76021, Karlsruhe, Germany

## Abstract

Neurogenesis in the developing central nervous system consists of the induction and proliferation of neural progenitor cells and their subsequent differentiation into mature neurons. External as well as internal cues orchestrate neurogenesis in a precise temporal and spatial way. In the last 20 years, the zebrafish has proven to be an excellent model organism to study neurogenesis in the embryo. Recently, this vertebrate has also become a model for the investigation of adult neurogenesis and neural regeneration. Here, we summarize the contributions of zebrafish in neural development and adult neurogenesis.

## Background

Neurogenesis describes the process by which undifferentiated neural progenitor cells generate mature and functional neurons. The first steps in neurogenesis are the induction of neural progenitors and a phase of cell division that enlarges the pool of progenitors. This is followed by a sequence of specification to committed progenitors, and differentiation to post-mitotic neurons. Each of these steps is spatially and temporally orchestrated to generate the multiple neuronal and glial cell types that will eventually populate the mature central nervous system (CNS). The zebrafish as a vertebrate model organism has been used in numerous studies of the various aspects of neurogenesis. Similar to mice and fruit flies, zebrafish can be used for genetic analysis. In contrast with mammals, the development of fish larvae occurs externally, making the zebrafish CNS accessible for experimental manipulation. In addition, such optical methods as high-speed and high-resolution microscopy, as well as new manipulative tools in the emerging field of optogenetics can be applied to this model organism. Thus, a unique combination of genetics, embryology, and state-of-the art optical techniques makes the zebrafish a unique vertebrate model organism to study neurogenesis.

The majority of studies conducted so far focus on neurogenesis at embryonic stages. However, recent studies have shown that the mature zebrafish brain may also serve as a valuable model for the study of adult neurogenesis. Indeed, as early as the 1960s, first experiments suggested that new neurons are born in the hippocampus and the olfactory bulb of the adult mammalian brain by *in-situ*^3^H]-thymidine labeling of newly synthesized DNA [1,2]. It took, however, more than 20 years before neurogenesis in the adult mammalian brain became widely accepted [3-7]. In rodents and non-human primates, the formation of new neurons is particularly evident in two regions, the subventricular zone (SVZ) and the subgranular zone (SGZ) of the telencephalon. However, neurogenesis has equally been reported in several brain regions outside the SGZ and SVZ [8], such as the basal forebrain [9], striatum [10,11], amygdala [12], substantia nigra [13], subcortical white matter [14], and, more recently, the hypothalamus [15-17]. Also the human brain seems to form neurons in the adult state [18-22]. The evidence for neurogenesis of olfactory bulb neurons derived from the SGZ in the adult human brain is, however, still controversial [23,24].

In contrast with mammals, teleosts like the zebrafish exhibit a much greater proliferative potential [25-27]. Up to 16 different proliferating regions were detected in discrete areas of the brain of adult zebrafish, including the regions equivalent to the mammalian SVZ and SGZ [26-30]. Around 6000 cells are born within every 30 min period in the adult zebrafish brain, representing ≈ 0.06% of the estimated 10^7^ cells of the zebrafish brain [31]. This widespread neurogenic activity has made the zebrafish an attractive model for the study of adult neurogenesis.

Here we summarize recent advances of neurogenesis in zebrafish both during development and in adulthood.

## Early neurogenesis

### Neural induction by extrinsic factors

The first step in the development of the vertebrate nervous system is the specification of the neuroectoderm. This process is called ‘neural induction’ and is initiated during early embryonic development. At the onset of gastrulation, the forming mesodermal layer involutes and comes into contact with the overlying ectoderm [32-34]. This presumptive mesodermal layer secretes important factors locally to induce or inhibit neural induction in the ectodermal layer.

Over the past years, important advances have been made showing that vertebrate neural induction relies on complex interactions between extrinsic signaling factors, such as members of the bone morphogenetic protein (BMP), wingless-integrated (Wnt) and fibroblast growth factor (Fgf) families [35,36], and the intrinsic transcription factor program, most importantly members of the SRY-box containing genes B1 (SoxB1) family [37-39]. According to the ‘default model’ described in studies in *Xenopus*, cells within the ectodermal layer have the tendency to differentiate into neural tissue [40]. Ventral secretion of BMPs, in particular BMP2, 4 and 7, blocks neural induction by inducing an epidermal fate [41,42]. BMP antagonists such as Noggin and Chordin are produced early in the dorsal pre-organizer region, which later forms the Spemann organizer, corresponding to the shield organizer in fish. These secreted proteins act permissively for the establishment of the neural fate in the dorsal ectoderm and allow the formation of the neural plate [32,33,42,43]. However, in rare cases low levels of BMP are required to maintain brain compartments such as protecting telencephalic fate in neural plate stage [44]. It turns out that inhibition of BMP signaling is required for acquisition of ‘neural plate’ fate but not sufficient [45]. Additional secreted molecules, such as Cerberus from the anterior endoderm [46] or Fgfs from the blastoderm margin [47-49] are needed to induce a complete neural fate. Indeed, Fgf signaling has been shown to induce a posterior neural ectodermal fate independent of BMP inhibition [50,51].

### Intrinsic factors determine the neuronal fate

**Figure 1:**
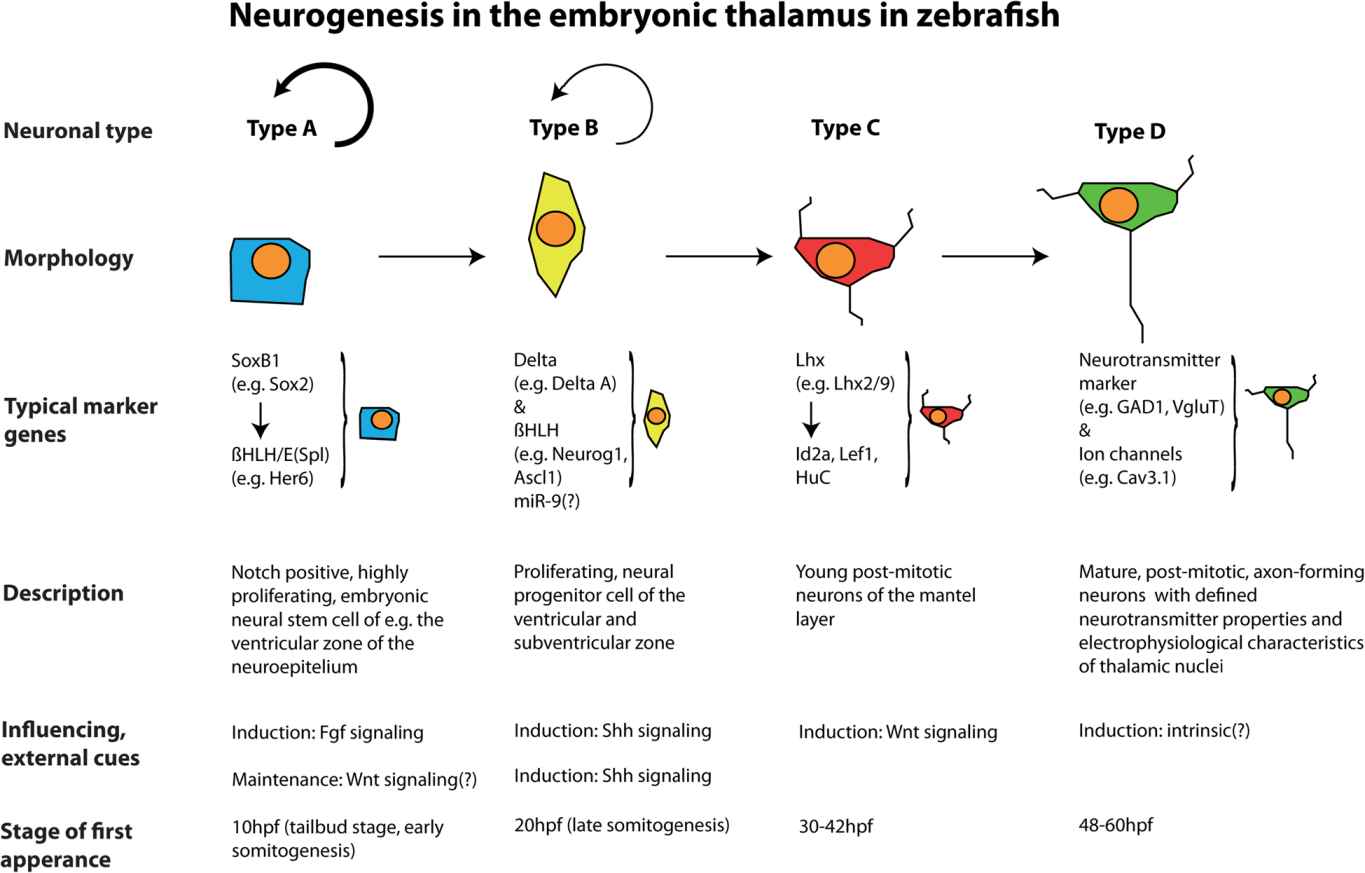
**Neurogenetic cascade in the embryonic thalamus of zebrafish.** Highly proliferating neural epithelial Type A cells are marked by *Notch*, *SoxB1* and *Her/Hes* expression. After downregulation of *SoxB1/Her/Hes*, thalamic progenitors become proneural Type B cells. Type C cells are immature, post-mitotic neurons, which express transcription factors, such as *lhx2b* and *lhx9*. Mature, post-mitotic Type B neurons can be identified by, for example, neurotransmitter-specific factors.

Recent data suggest that members of the SRY-box containing genes *B1* (*SoxB1*) gene family are the transcriptional basis of this ‘default program’: The *SoxB1* genes are important for specification of the embryonic ectoderm into Type A neuroectodermal lineage in vertebrates [38,39] (Figure 1).

The neural ectoderm is specified by members of the *SoxB1* family [52-56]. So far, the following members of the *SoxB1* have been characterized in zebrafish: *sox1*(*a*/*b*), *2*, *3*, and *19*(*a*/*b*). All of these are induced during blastula stages and are redundantly required to specify neural ectodermal fate [57,58]. In fact, *sox2* can be found in neural progenitors in the embryo as well as in neural stem cells in the adult zebrafish brain [29,30,58,59]. In combination with Pou2/Oct4, Sox2 activates repressors of neuronal differentiation, such as *hesx1* and *her3*[58,60]. Hence, Sox2 is one of the most important factors required for the maintenance of neural progenitor properties and functions in the vertebrate lineage [61-63]. Prior to differentiation, *SoxB1* members become downregulated, so that they are no longer expressed, neither in immature neurons nor in post-mitotic terminal neurons [39,64]. In addition, in the adult zebrafish brain, *SoxB1* members label quiescent and proliferating glia as well as juvenile neurons.

However, one cannot entirely separate extrinsic cues from intrinsic factors. In zebrafish, the expression of the *SoxB1* family member *sox3* depends on early Fgf signaling from the blastoderm margin [50] and, in turn, regulates expression of early BMPs, such as BMP2 and 7 [58]. In summary, this suggests that in concert with the inhibition of BMP signaling and activation of Fgf signaling, *SoxB1* members are important factors for maintaining the pool of neural stem cells in early gastrulation stages in the zebrafish embryo.

### From neural plate to neural tube

Once specified, the neural ectoderm forms the neural plate, a pseudostratified epithelial structure in zebrafish. During early somitogenesis in fish, the neural plate converges and forms the neural keel, eventually fusing at the dorsal midline to form the neural rod. Starting from the anterior, cells in the emerging neural keel become polarized at the embryonic midline. A recent study suggests that midline polarization of structural proteins, such as Pard3 and Rab11a, occurs during cell interdigitation in the neural rod [65]. The subsequent apical localization of these proteins, including Zona Occludens protein ZO-1, aPKC, and the basolateral localization of Numb and Lgl2 and the subsequent mirror-symmetric cell divisions in the medial neural rod lead to the formation of its lumen, the neurocoel, which will give rise to the brain ventricles and is important for the expansion neural progenitor pool [66-68].

During the process of neurulation, the zebrafish differs from other vertebrates: instead of folding up the neural plate immediately into a tube with a lumen, it first forms a solid neural keel. However, the topological arrangement of cells in zebrafish during formation of the neural keel from the neural plate is similar to that of other vertebrates. In the so-called ‘secondary neurulation’, the fish neural rod inflates and forms a vertebrate typical tube. Thus, although there are differences, neurulation in fish and mammals leads to the formation of a highly similar structure in each case, the neural tube [69].

After lumen formation, we find an increasing number of asymmetrically dividing cells in the neural tube. The asymmetric inheritance of a subcellular membrane domain of the dividing progenitors is strongly correlated with the asymmetric fate of the daughter cells [70]. A more apically derived daughter cell becomes the neuron, whereas a more basal daughter replenishes the apical progenitor pool. It was suggested that Notch signaling is important for the fate decision: basal self-renewing daughter cells display high Notch activity and an apical differentiating daughter cell show a low Notch activity [71]. The directionality of Notch signaling involves Pard3-dependent asymmetric localization of Mindbomb to the apical daughter.

The dividing neuronal progenitors move from the apical side of the neural epithelium to the pial side in a process described as ‘interkinetic nuclear migration’ (reviewed in [72]). Cells divide at the apical side and differentiate in the basal zone. Experiments in the zebrafish retina have shown that if migration is speeded up and cells remain too long in the basal zone, progenitors exit the cell cycle prematurely. This leads to an increase of early-born projection neurons at the expense of late-born interneurons [73]. Thus, the establishment of apical-basal polarity of neuroepithelial cells is critical for CNS maturation.

### Initiation of neurogenesis by bHLH factors, the Enhancer-of-split E(Spl) subfamily

The onset of neurogenesis in the zebrafish neural plate becomes apparent during late gastrulation by the expression of proneural genes [74]. The first proneural genes expressed encode transcription factors, such as the bHLH genes *neurogenin1* (*neurog1*; [75,76]) and *achaete-scute1* (*asc1*, Figure 1) [77]. Transcription of these genes is not ubiquitous but restricted to cell clusters that are Type B proneural clusters [75], from which the nuclei of the primary neuronal network will arise [78].

At this stage, one can still find large *SoxB1* positive, non-neurogenic territories that separate the first proneural clusters (reviewed in [79]). These domains will be progressively recruited towards their neural fate during early neurogenesis. Members of the E(Spl) family, the Her/Hes genes, are transcriptional targets of *SoxB1*, that is, *her3*[58], and are expressed in various combinations in most domains of non-neurogenic territories, such as the other *hairy* genes *her5*, *her6*, *her9*, and *her11*[80,81]. Ectopic expression of these *Her/Hes* genes lead to a downregulation of *neurog1* expression whereas loss-of-function experiments show an increase in *neurog1* expression.

The function of *hairy* genes, for example, Her5, has been studied intensively during midbrain-hindbrain boundary (MHB) formation [79,82]. The progenitor pool at the MHB is located between the ventrocaudal cluster of the midbrain and presumptive neurons of anterior rhombomeres. At the onset of neurogenesis, cells of this progenitor pool give rise to differentiated cells within the MHB domain [80]. This progenitor pool is marked by *her5* and *her11* expression and combined blockage of these E(Spl) factors suggests that they are partially redundant in domains of co-expression [83-85]. Both factors block expression of several proneural genes such as *neurog1*, *ascl1a*, and cyclin-dependent kinase inhibitors, but their direct transcriptional targets remain unknown.

Taken together, SoxB1/Hairy-dependent, active inhibition of neurogenesis is the crucial mechanism expanding the pool of cells. Spatially controlled downregulation of *Hairy* gene activity defines progenitor pools within the zebrafish embryonic neural plate.

According to their dependency on active Notch signaling, *hairy* genes can be grouped into two families [86]. The expression of the *Her* genes mentioned above, *her3, her5, her6, her9* and *her11* are independent of Notch signaling [83,87]. This is in contrast with the important function of other hairy factors, such as *her4*. Within each cluster, cells expressing higher levels of proneural genes are selected as ‘neuroblasts’ for further commitment and differentiation, while concomitantly maintaining their neighbors as proliferating neural precursors available for a later round of neuroblast selection [88]. This process of ‘lateral inhibition’ relies on Notch-dependent Her4 signaling in precursors, and expression of the bHLH transcription factor Delta in the neighboring neuroblasts [89,90]. Her4 is required to maintain neural progenitor fate in the embryo as well as neural stem cell fate in later stages [91-93] The *her4-*positive proliferating neural precursors can be found within proneural clusters and fulfill an important function during the selection of neuronal versus glial fate by lateral inhibition [89].

### Early anterior-posterior patterning of the neural plate

One of the most fascinating problems in developmental biology is how positional information is assigned along the anterior-posterior (AP) axis, especially in the neuroectoderm. Following neural induction, progenitor cells display an anterior neural plate fate and subsequently become transformed to adopt a more posterior character by signals originating from more caudal regions of the embryo [94,95]. Among the postulated posteriorizing signals in zebrafish are Wnt, Fgf and Nodal proteins, as well as retinoic acid [96,97]. Wnt proteins are likely to be the earliest patterning signals acting in the forming neural plate primordium. The zebrafish mutants *headless* (*hdl*) and *masterblind* (*mbl*) carry mutations in the genes encoding *tcf3* and *axin1*, respectively, and exhibit severe anterior patterning defects [98-100]. In these embryos, the forebrain and the eye anlage are reduced or absent and the midbrain expands forward to the tip of the neural tube. Recent studies have shown that Wnt8, expressed in the non-axial mesendoderm, is important for acquisition of hindbrain fate [101]. Consistent with this, Wnt8 is required for induction of the hindbrain expression of *gbx1 *[102]. To establish a Wnt-gradient, a counteracting signal must be released from the most anterior neural structures. The anterior neural boundary (ANB) indeed acts as an organizer to pattern the anterior neural plate by the release of secreted frizzled-related protein (sFRP) Wnt antagonists, such as TLC [103,104]. Thus, graded Wnt activity acts to initiate AP patterning within the neural plate. Following this initial patterning step, further mechanisms maintain and refine regional identity in the neural tube, for example, the formation of local boundaries such as between the forebrain and midbrain [105]. These boundaries often co-localize, with local organizing centers within the neural tube mediating specific positional information along the AP axis from anterior to posterior [106,107].

One of these local signaling centers is the mid-diencephalic organizer (MDO), which is established at the intrathalamic boundary, the zona limitans intrathalamica [108]. The MDO is characterized by *shh* expression [109-112] and orchestrates the development of the thalamic complex in the diencephalon in zebrafish [113]. *shh* acts as the principal signal of this organizer and is required for the concentration-dependent induction of *neurog1* and *ascl1*, leading to the establishment of the thalamic nuclei in vertebrates [81,114].

Within the neural tube, a further well-characterized organizing center is the MHB organizer at the isthmus rhombencephali (reviewed in [115,116]). The MHB is able to induce cellular fates in a nonautonomous manner by the secretion of the principal signal Fgf8 [117,118].

Initially, other signaling factors, such as the canonical Wnt proteins Wnt1, Wnt3, Wnt3a, and Wnt10b, are broadly expressed throughout the forebrain and midbrain, until their expressions become restricted to the MDO, the MHB, and the dorsal midline [119,120]. Wnts do not elicit inductive effects comparable to those of the principal signals Shh and Fgf8. However, blockage of Wnt signaling leads to a lack of local organizing centers [120,121]. Thus, Wnt signaling activity is a common theme upstream of many local brain organizers [113].

### The thalamus in zebrafish – an example for controlled neurogenesis

Vertebrate neurogenesis within a proneural cluster is regulated with a high degree of temporal and spatial precision, with stereotypic patterns of neuronal differentiation and extensive neuronal migration [122,123]. Dynamic patterns of mitotically active neuronal precursors within the neuronal clusters have been described as ‘neurogenetic gradients’ [124]. In the thalamus, the progression of neuronal differentiation from posterior to anterior presents such a neurogenetic gradient [108]. Recently, we could show that this mechanism is used to guide neurogenesis in the developing caudal diencephalon, that is, the thalamus [81]. The thalamic primordium is initially a non-neurogenic territory. Shh from the MDO determines the neurogenic program of the thalamus by inducing proneural genes such as *neurog1*. However, neurogenesis does not start immediately after induction of Shh expression in the thalamic complex and is first suppressed [81]. E(Spl) factor-positive domains separate the proneural clusters. These domains exhibit delayed differentiation and become progressively neurogenic only in later developmental stages (reviewed in [79]). Neurogenesis in the thalamic complex is regulated by the E(Spl) factor Her6 [81]. The widespread expression of *her6* in the anterior neural plate represses the Neurog1-mediated neurogenesis cell-autonomously. The subsequent posterior-to-anterior regression of *her6* expression is accompanied by up-regulation of *deltaA* and *neurog1* in the ventricular and subventricular zones of the neural tube in cells that formerly expressed *her6*. The mechanism of Her6 regression is not well understood; however, different explanations have recently been proposed. Firstly, Shh has been suggested to influence the stability of E(Spl) factors, such as Hes1, the murine homolog of Her6 [125], which could explain the maintenance of *her6* expression in the vicinity of the MDO. Secondly, microRNAs have been reported to play an important role in the progression of progenitors to post-mitotic neurons [126]. Indeed, microRNA-9 defines an intermediate, ambivalent state by repression of both the progenitor marker Her6 as well as the post-mitotic marker Elavl3, formerly known as HuC. Thus, miR-9 facilitates the transition of progenitors toward cell-cycle exit at late stages of embryonic development [127], to enable the nervous system to generate an increased number of neurons at this stage [128]. Indeed, the dynamic of the Her6 regression from posterior to anterior is later reflected in the induction of *neurog1* expression and the generation of Type C, post-mitotic thalamic neuronal markers such as *id2a, lhx2b, lhx9* and *HuC *[129] (Figure 1). These transcription factors orchestrate the subsequent maturation to Type D neurons with defined neurotransmitter properties. Hence, a crucial mechanism that regulates neurogenetic gradients within the embryonic CNS is a process of spatially controlled de-repression of neurogenesis, mediated by the local inactivation of the Her/Hes proteins.

## Adult neurogenesis

### Neurogenesis in the adult zebrafish telencephalon

**Figure 2:**
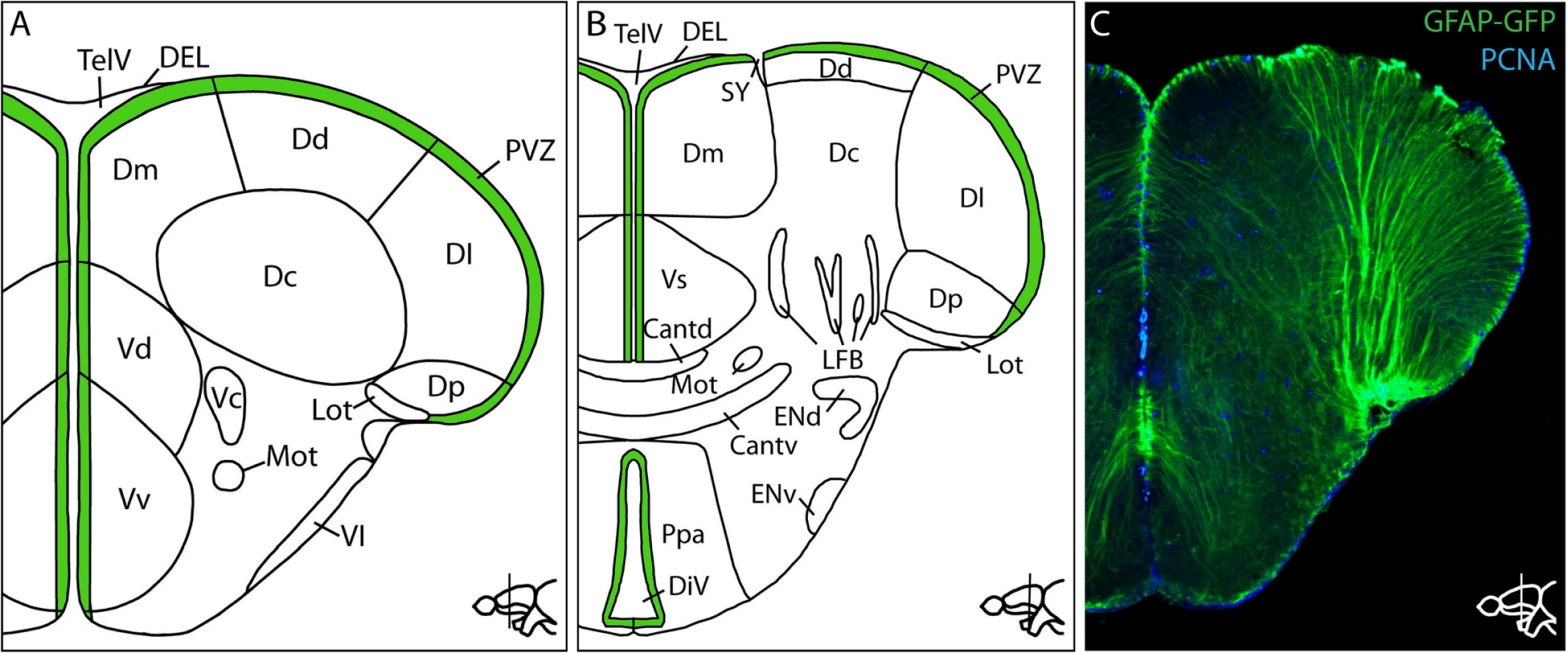
**Anatomy of the telencephalon of the adult zebrafish.** Schematic of transverse sections through the anterior (**A**) and posterior (**B**) zebrafish telencephalon indicating the different anatomical subdomains. Proliferative periventricular zones are indicated in green. Schematic was redrawn from [191]. **C**: Radial glial cells have long processes that reach all the way to the pial surface. Immunostaining against GFP in the *Tg*(*GFAPGFP*) line marks the radial glial cells in the telencephalon of an adult zebrafish. The section was co-stained with the proliferation marker PCNA (proliferating cell nuclear antigen) to distinguish between PCNA-negative Type I and PCNA-positive Type II cells (described in [30]). Cantd: commissural anterior, pars dorsalis; Cantv: commissural anterior, pars ventralis; Dc: central zone of the dorsal telencephalic area (D); Dd: dorsal zone of D; DEL: dorsal ependymal lining; DiV: diencephalic ventricle; Dl: lateral zone of D; Dm: medial zone of D; Dp: posterior zone of D; Lot: lateral olfactory tract; ENd: entopeduncular nucleus, dorsal part; ENv: entopeduncular nucleus, ventral part; LFB: lateral forebrain bundle; Mot: medial olfactory tract; Ppa: anterior part of parvocellular preoptic nucleus; PVZ: periventricular zone; SY: sulcus ypsiloniformis; TelV: telencephalic ventricle; Vc: ventral nucleus of the ventral telencephalic area (V); Vd: dorsal nucleus of V; Vl: lateral nucleus of V; Vp: postcommissural nucleus of V; Vs: supracommisural nucleus of V; Vv: ventral nucleus of V.

The mammalian telencephalon develops by evagination of the neural tube. In contrast, the telencephalon of teleosts forms by outward folding (eversion) so that the proliferative periventricular zones are not only present internally but also cover the outer surface of the telencephalon [130-133] (see Figure 2). Proliferative zones form discrete areas that differ in the architecture of the stem cell niche [134]. In mammals, the ventricular surface is covered by an epithelium, the so-called ependyma or ependymal layer [135-138]. In zebrafish, the ependymal layer is restricted to the roof of the telencephalic ventricle and to dorsolateral regions of the proliferative zones (D and Dm; see Figure 2) [134]. The other proliferative zones are devoid of an ependymal layer. In this respect, the cellular environment of the zebrafish stem cell niche resembles more closely the fragmented ependymal lining of the stem cell niches of reptiles and birds (reviewed in [139]). Proliferative areas also differ with respect to the presence of ciliated cell types. The D, Vd, Dm, and Dl areas (Figure 2) contain multiciliated cells whereas the stem cells at the ventricular cavity in the Vv are devoid of cilia [134]. Furthermore, proliferation is modulated along the rostrocaudal axis of the telencephalon: While cell proliferation can be detected in the entire periventricular zone at rostral levels, proliferation is confined to distinct subdomains in more caudal areas of the telencephalon [134].

Proliferative zones differ also in their rate of formation of new cells [26]. The most proliferative region is found in the medial subpallium (Vv) [26,30,140]. This region, in contrast to the other proliferative zones, consists almost exclusively of bromodeoxyuridine-positive (BrdU), S100 calcium-binding protein β-negative (S100β), non-ciliated cells, with neurons forming the deeper layer of the niche. In contrast with the other stem cell niches, no quiescent cells are present in this region [30,134]. Furthermore, only cells in the very dorsal part of Vv express S100β. The region is homologous to the SVZ of mammals and generates mostly neurons that migrate via the rostral migratory stream into the olfactory bulb [26,27,141-143]. In contrast with mammals, however, this region also generates neurons that settle in the adjacent parenchyma [26,141]. Lower proliferative rates were scored in other ventricular regions with somewhat elevated levels in the posterior zone of the dorsal telencephalic area (Dp), equivalent to the SGZ of the mammalian hippocampus [25,27]. Some proliferating cells were also detected throughout the parenchyma [26,144]. Marker analysis showed that these parenchymal cells are dividing oligodendrocyte precursor cells (OPCs) that both self-propagate and give rise to mature oligodendrocytes [144].

Cellular key players in adult zebrafish neurogenesis are radial glia cells. These cells, whose cell bodies are located close to the ventricle, have long processes that reach all the way to the pial surface of the telencephalon, where they terminate with their endfeet on the walls of blood vessels or at the pial surface itself [59,134].

**Figure 3:**
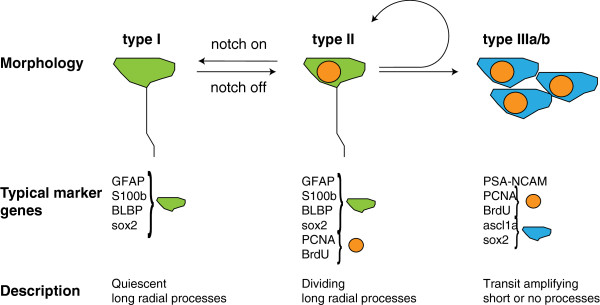
**The major proliferative cell types in the telencephalon stem cell niche.** The markers expressed by the three major cell types are indicated [30]. Notch signaling transforms proliferating Type II radial glial cells into quiescent Type I radial glial cells [151]. Note that there exists heterogeneity in the cellular composition and marker expression of stem cell niches in the telencephalon [30,140].

Radial glial cells in the adult zebrafish telencephalon proliferate and, in addition to self-renewal, are able to generate new neurons [145]. Radial glial cells have also been observed in the developing mammalian telencephalon, where they act as embryonic neural stem cells. Mammalian radial glia disappear shortly after birth, giving rise to ependymal cells and astrocytes, some of which retain stem cell potential in the SVZ and SGZ [146-148]. Thus, the adult zebrafish appears to have retained embryonic features. Indeed, the adult zebrafish radial glial cells express similar genes to the embryonic counterparts, such as the glial acidic fibrillar protein (GFAP), S100β and the brain lipid-binding protein (BLBP) [26,59,149,150]. With the expression of proliferation markers, such as PCNA, or by detecting incorporation of the thymidine analog BrdU, one can distinguish two classes of radial glia cells: those which are in a quiescent state (Type I cells, BrdU−, PCNA−, GFAP+, S100β+, BLBP+) and slowly cycling cells (Type II cells, BrdU+, PCNA+, GFAP+, S100β+, BLBP+). Furthermore, *Her/Hes* genes, such as *her3*, *her4*, *her5*, *her6*, *her8a*, and *her15*, generally characterize quiescent rather than actively proliferating radial glial cells [93,140]. The majority of radial glial cells are in a resting state. Type II cells give rise to neuroblasts (Type III cells) that continue to proliferate, turn on neural marker genes, such as *PSA-NCAM* and the proneural gene *ascl1*, and eventually enter the rostral migratory stream or leave the periventricular zone to move deeper into the parenchyma. Proliferating Type II stem cells can divide symmetrically to self-renew or asymmetrically to generate Type III cells [145]. The relative proportion of proliferating and resting stem cells can be shifted by interfering with Notch signaling: Activation of the Notch pathway drives stem cells into quiescence, suggesting that cross talk between cells controls the maintenance of the quiescent stem cell population [151] (Figure 3). Hence, maintenance of neural stem cells in the adult appears to employ a similar mechanism as that seen in the embryo, where Notch-mediated lateral inhibition maintains a pool of embryonic neural stem cells for later production of neurons, such as the secondary motor neurons [152]. In the periventricular domain (Vv in Figure 2), but not in dorsal proliferative domains, Fgf signals are regulators of cell proliferation [140]. This highlights the differences of the stem cell niches in the ventral and dorsal telencephalon and points to Fgf signals as potential causes of the higher proliferation rate in the ventral proliferative zone.

The long processes of radial glial cells have been proposed to serve as a scaffold for newborn neurons to migrate out of the ventricular zone [59,149,150]. However, most newborn neurons appear to settle in subperiventricular areas [92], suggesting that the majority of newborn neurons will not migrate to the pial surface as many types of newborn neurons do in the embryonic brains of fish [129] or mouse [153]. The cellular architectures of the stem cell niches are more complex than summarized in the schematic of Figure 3. For example, in addition to S100β/glutamine synthetase (GS)/GFAP/aromatase B-positive radial glial cells also S100β/GS/aromatase B-positive but GFAP negative cells were found at a smaller proportion in the dorsal and dorsolateral regions [30,140]. Moreover, the electron microscopic study of [134] detected additional cell types that differ in cellular morphology and marker expression. The Type IIa cells reported in [134] have shorter processes than the radial glia cells (the Type I and II cells described by [30]) and contact blood vessels in the vicinity of the stem cell niche [134]. This suggests the existence of additional cell types that may be involved in neurogenesis, either directly by being specialized progenitors or indirectly by contributing to the architecture of the niche.

### Other neurogenic sites in the adult zebrafish brain

While the stem cell niches of the adult telencephalon are the most intensively investigated, several studies also reported on other proliferative regions in the brain. The cerebellum shows highly abundant neurogenesis [27]. The dividing cerebellar neural progenitors express the progenitor markers nestin, Sox2, Meis homeobox 2 (Meis) and Musashi homolog 1 (*Drosophila*) (Msi1). They also exhibit neuroepithelial properties such as expression of ZO-1, β-catenin, γ-tubulin, and protein kinase C (aPKC) [29]. These dividing cells do not express radial glial markers. The cerebellar niche, however, also contains radial glia-like cells expressing GFAP, vimentin, and BLBP. These cells appear to act as scaffolds for migrating progenitor cells rather than as stem cells *per se*. As found in the periventricular Vv, Fgf signaling is an important regulator of stem cell activity in the cerebellum. Blocking Fgf signaling with a heat-shock inducible dominant-negative Fgf receptor leads to a significant reduction of proliferating cells [29].

Another neurogenic site is located at the boundary between the midbrain and the hindbrain [86]. This region expresses the *her5:gfp* transgene in cells that form a restricted cluster at the ventricle of the MHB. These *her5:gfp* positive cells fulfill several general hallmarks of neural stem cells. A subpopulation of the *her5:gfp* cells cycle slowly, express the stem cell markers GFAP, BLBP, Sox2 and Musashi, and can differentiate into neurons and glia [86]. *her5:gfp*-expressing cells resemble the Type II cells of the telencephalic niche. However, evidence for the presence of an intermediate fast proliferating cell type such as the transit amplifying cells of the mammalian stem cell niches or the Type III cells of the telencephalic niches of the zebrafish brain is lacking.

In the optic tectum of adult zebrafish, proliferating cells exist in the medial, lateral, and caudal margins of the PGZ [25,27,154-156]. Proliferating cells in these regions express the neural progenitor markers PCNA, Sox2, Msi1 [157-159]. These cells do not express glial markers. However, they show neuroepithelial characteristics, such as the expression of ZO-1, γ-tubulin, and aPKC [73,160]. BrdU lineage tracing showed that these cells can differentiate into glutamatergic or GABAergic neurons, oligodendrocytes and radial glia, and hence represent a multipotent progenitor pool [156,161-163]. Similar to the architecture of the cerebellar stem cell niche, non-dividing radial glia-like cells expressing glial markers (GFAP, BLBP, S100β) reside adjacent to the proliferating neuroepithelial-like progenitors. In addition, proliferating radial glia-like cells can be detected in the deeper layer of the PGZ [156].

Although the analysis of these stem cell niches in comparison to the telencephalic niches is more limited, results suggest that the architecture of the different stem cell niches in the zebrafish brain differ significantly, which may reflect differences in the neurogenic potential of the sites.

### Regeneration of the adult brain

Regeneration in the mammalian brain is very limited. It seems that the local environment in most parts of the mammalian brain is not suited for the long-term survival of newly formed neurons [164,165]. In contrast, zebrafish have a remarkable ability to regenerate injured organs, including the CNS [166-170]. Zebrafish regenerate axons [171] and whole neuronal assemblies with full restoration of function after injury [28,172-180].

**Figure 4:**
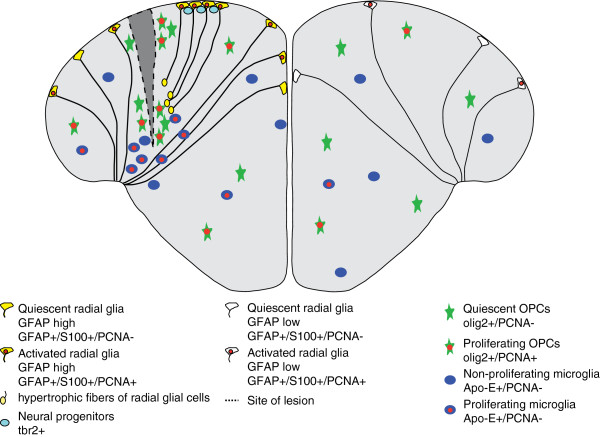
**Regenerative responses to stab wound injury.** Lesions introduced by a syringe needle induce a proliferative response in the periventricular region of the stabbed hemisphere. Oligodendrocytes and microglial cells accumulate at the site of lesion. The glial marker GFAP and the proliferation marker PCNA are upregulated in the lesioned hemisphere. The number of *T-box brain gene 2-positive* (*Tbr2+*) cells is increased upon injury [179]. The injury is totally healed after 30 days without traces of a glial scar. Schematic shows transverse section through the medial telencephalon.

After wounding the telencephalon by stabbing with a needle, neurogenesis is stimulated along the entire ventricular surface (Figure 4), with many more Type II stem cells expressing the proliferation marker PCNA within 3 days after wounding [179]. This proliferation response is restricted to the injured hemisphere and persists at least up to 14 days [92,179]. In contrast with the predominant settlement of newborn neurons at the subventricular zone during constitutive neurogenesis, newborn neurons in the injured brain migrate over longer distances into the area of damaged tissue and differentiate into mature neurons [92,180]. Precisely which long-range signals mediate the increase of proliferation in the periventricular stem cell zones even at sites distal to the lesion remains unknown as yet. Chemokine signaling via Cxcr5 may play a role in this process [181]. It is also unclear why the activation remains restricted to the lesioned hemisphere [179], even though the proliferative zones in the medial telencephalon are separated only by a narrow ventricular gap.

Some proliferation is also detectable at the site of lesion, which has rapidly filled with blood cells after the injury. However, these proliferating cells are mostly microglia involved in removal of the cellular debris [92,179,180]. In addition, OPCs accumulate at the site of lesion. These OPCs proliferate only to a limited extend and, hence, most of them appear to have migrated in from adjacent regions [179]. The accumulation of microglia and OPCs at the site of lesion is transient [179]. There is no scar formation in the zebrafish brain, as is seen in injured mammalian neural tissue [92,179,180]. In mammals, glial scars are thought to act as mechanical and biochemical barriers that prevent ingrowth of new axons or migration of cells into the lesion site [182]. By three weeks after injury, the proliferation rate at the ventricular zone has returned to the base line seen in uninjured brains, and the damaged tissue has recovered totally, without any signs of the traumatic impact remaining [179,180]. Interestingly, the molecular mechanism underlying constitutive and reactive neurogenesis in response to injury seems to differ: reactive neurogenesis depends on the zinc-finger transcription factor gata3, which is not expressed in the uninjured brain [183,184].

A fundamental question is why fish have this extraordinary ability to regenerate adult neural tissues. Zebrafish also grow in adulthood, with generation of new neurons that need to be incorporated into existing neuronal circuits, suggesting that this generates a favorable physiological situation for the repair of injured neural tissue. In lesions of the adult fish cerebellum, damaged cells seem to be removed by apoptosis [185]. In contrast, necrotic cell death is predominant in injured mammalian neural tissue [186,187]. Necrosis triggers inflammatory responses much more strongly than apoptosis, gradually leading to loss of neural cells and scar formation [188]. Thus, the difference in the mode of cell death may be one of the factors that contribute to the extraordinary recovery of neural tissue in fish after injury. The oligodendrocyte marker Olig2, which suppresses neurogenesis in mammals [189], is massively upregulated in injured mammalian neural tissues. By contrast, in the zebrafish telencephalon, *olig2:EGFP*-expressing cells accumulate transiently at the site of lesion [179]. It was demonstrated recently that inflammatory signals trigger reactive neurogenesis in the zebrafish telencephalon, pointing at yet another fundamental difference in the regenerative responses in teleost and mammal brains [190]. The central questions are whether we can learn from zebrafish how, for example, scar formation is suppressed and what the causes of the remarkable plasticity of neural tissue are in the zebrafish. Most of all, a burning challenge will be to use this knowledge from the fish brain to modulate the behavior of stem cells, neurons, glia, and immune cells in the injured human brain.

## Conclusion

The regulatory cascades in the embryo and adult have similarities in the sequence of events underlying formation of functional neurons. However, the tissue settings are fundamentally different. In the embryo, neurons are derived from the neuroectodermal epithelium, which becomes structured in a hierarchy of increasing complexity. In contrast, adult neurons originate mainly from glia cells and need to be incorporated into a preexisting, fully functional tissue. Moreover, in case of repair, immune responses, cell death in addition to the complexity of a mature tissue are factors that contribute to the successful restoration of structure and function. This may explain the usage of different genetic regulators in the cascades. Elucidating these specific differences will provide additional insights into how neurogenesis is mediated in the embryo and adult. Detailed knowledge will enable us to develop tools that might be used to increase the neurogenic potential of neural stem cells in terms of their ability to undergo neuronal determination and differentiation. In the future, these insights may form the basis for the design of therapeutic strategies that will allow eventually the activation of quiescent progenitors and their successful incorporation in functional neuronal circuits in the mammalian brain.

## Abbreviations

ANB: Anterior neural boundary; AP: anterior-posterior; BLBP: Brain lipid-binding protein; BMP: Bone morphogenetic protein; BrdU: Bromodeoxyuridine; CNS: Central nervous system; Fgf: Fibroblast growth factor; GFAP: Glial acidic fibrillar protein; KIT: Karlsruhe Institute of Technology; MDO: Mid-diencephalic organizer; MHB: Midbrain-hindbrain boundary; OPC: Oligodendrocyte precursor cells; PCNA: Proliferating cell nuclear antigen; sFRP: Secreted frizzled-related protein; SGZ: Subgranular zone; SVZ: Subventricular zone; Wnt: Wingless-integrated.

## Competing interests

The authors declare that they have no competing interests.

## Authors’ contributions

SS summarized neurogenesis in the embryo, whereas RS and US focused on neurogenesis in the adult. All authors read and approved the final manuscript.
